# Effect of plant edges strips on the conservation soil properties in modern farming field

**DOI:** 10.1371/journal.pone.0299104

**Published:** 2024-04-16

**Authors:** Lina Šarūnaitė, Aušra Arlauskienė, Danutė Jablonskytė-Raščė

**Affiliations:** Lithuanian Research Centre for Agriculture and Forestry, Akademija, Kėdainiai District, Lithuania; University of Minnesota, UNITED STATES

## Abstract

The European Green Deal encourages the use of non-productive activities in agriculture. One of the measures is the cultivation of melliferous floral plants at the field margins. Their influence on soil compaction and other deterioration is due to heavy machinery, its inappropriate use and frequent driving on field margins, is little studied. Plants of a high environmental value though rarely grown by farmers were selected for melliferous plant strips: perennial grass swards (PGS), perennial legume swards (PLS), annual floral plats mixture (AEP) and natural grassland swards (NGS). The experiment was installed on a clay loam and loam *Cambisol* with the aim to determine the effect of different plant composition strips grown at the field edges on the physical and chemical soils parameters of with different granulometric structure. It was found that the highest amounts of roots and plant residues in the soil were left after cultivating sward strips of PGS and NGS compared to the field where cereals had been intensively grown. The amounts of root and plant residues produced by plants, soil rest increase the amount of organic carbon in the soil. During the five-year period, the plants edges strips improved the properties of the field margin top and subsoil.

## Introduction

The high additive value agricultural production encourages the farms enlargement, narrowing specialization, and modernization of the farm’s technical base. However, heavy tractors, agricultural equipment and their inappropriate use can have negative effects on the soil (compaction, structure, organic matter, water and mechanical soil erosion etc.), especially at the field’s edges. Also, physical and chemical degradation of the soil is enhanced biological reasons: lack of crop diversification in the crop rotation, abundant use of mineral fertilizers and pesticides, which results in low organic matter content and limited diversity of biota [1–3]. Processes can be uncontrolled in the interaction of soil properties, plants, and climate [4]. The compaction of fields edges is the result of heavy machinery weight with narrow tires, when agricultural machinery drives several times thought the same place. The heavy machinery loads reach deep layer of the subsoil, therefore increasing the risk of subsoil compaction [5]. Soil deformation under integrated systems depends on many factors and one of them the compression level [6]. The soil at the edge of the field is compressed, scores appear, the seed bed is poorly prepared, and the plants germination is not homogeneous. In such a soil, organic matter is oxidized and decomposed, the soil agrophysical properties deteriorated which complicates the availability and assimilation of nutrients for plants [7]. This is particularly the case in soils with severe granulometry, where high levels of clay particles are responsible for the negative properties of this soil [4]. The consequence of this process is the empty and weedy edges of the agricultural fields as well as the lower yield of the crops, increased energy, time, and cost.

Integrating perennial swards into crop rotations, adopting new cropping techniques and identifying ecological services can help solve these problems. Natural plant communities play many important functions in the agricultural landscape [8] by very rich in their floristic diversity [9] and improvement the soil structure by plant roots development, penetration, volume, nutrient uptake [10,11]. Soils with layers that are not peeled and too often unreachable are rich in soil active organisms and their diversity. Living in the soil bacteria, fungi, protozoa break down the vegetable residues into the soil, some of which is converted into the other part of nutrients, and other part joined into humus material composition [12]. Biological processes include actions by earthworms’ which forming cavities and large pores in the soil that move water and air [13]. Also, modern agricultural intensification, increased fertilizer and pesticide use will inevitably contribute to the loss of semi-natural habitats. Many studies, meta-analysis resulted with the conclusions that flower strips created at arable field edges increases pest control and pollination by providing forage, shelter, and nesting places by creating pollinator communities [8,14–17]. Reducing mechanical impact on soil creates a durable soil structure that provides optimum air and water regime, nutrient availability for plants, and soil biota [18].

The "green architecture" of the EU’s new Common Agricultural Policy encourages the voluntary implementation of practices beneficial to the climate and the environment—various non-productive activities. One of them is the flowering plant strips on field edges. We hypothesize that the establishment and cultivation of perennial flowering swards strips over a longer period may contribute to restoring and improving the soil properties of field edges. A very important ecological service is to attract pollinator insects in intensive farming conditions, however, to improve soil parameters in the field edges is less studied. The aim of this study was to determine the effect of different flowering annual and perennial herbaceous plants strips grown at the edges of fields on the physical and chemical parameters of soils with different granulometric composition.

## Materials and methods

### Experimental sites

The experiment was carried out during 2013–2018 at two experimental sites of Lithuanian Research Centre for Agriculture and Forestry. The first site was established at the Joniškėlis Experimental Station on a clay loam *Endocalcaric Endogleyic Cambisol* (Clayic, Drainic) (WRB, 2014), the second–at the Institute of Agriculture in Akademija, Kėdainiai district on a loam *Endocalcaric Epigleyic Cambisol* (Drainic, Loamic) (WRB, 2014). The soil texture of Joniškėlis site is clay loam (at a depth of 0–25 cm) on silty clay (at a depth of 26–76 cm), with deeper lying sandy loam (at a depth of 77–135 cm). The topsoil layer (0–25 cm) contains 27.0, 50.2 and 22.7% clay, silt and sand, respectively. Bulk density of the ploughlayer is 1.5 Mg m^-3^, total porosity 41–43%. At Akademija site the topsoil layer (0–25 cm) contains 19 clay particles, silt– 29 and sand 52%. Bulk density of ploughlayer was 1.6 Mg m^-3^, total porosity– 43–44%.

**Fig 1:**
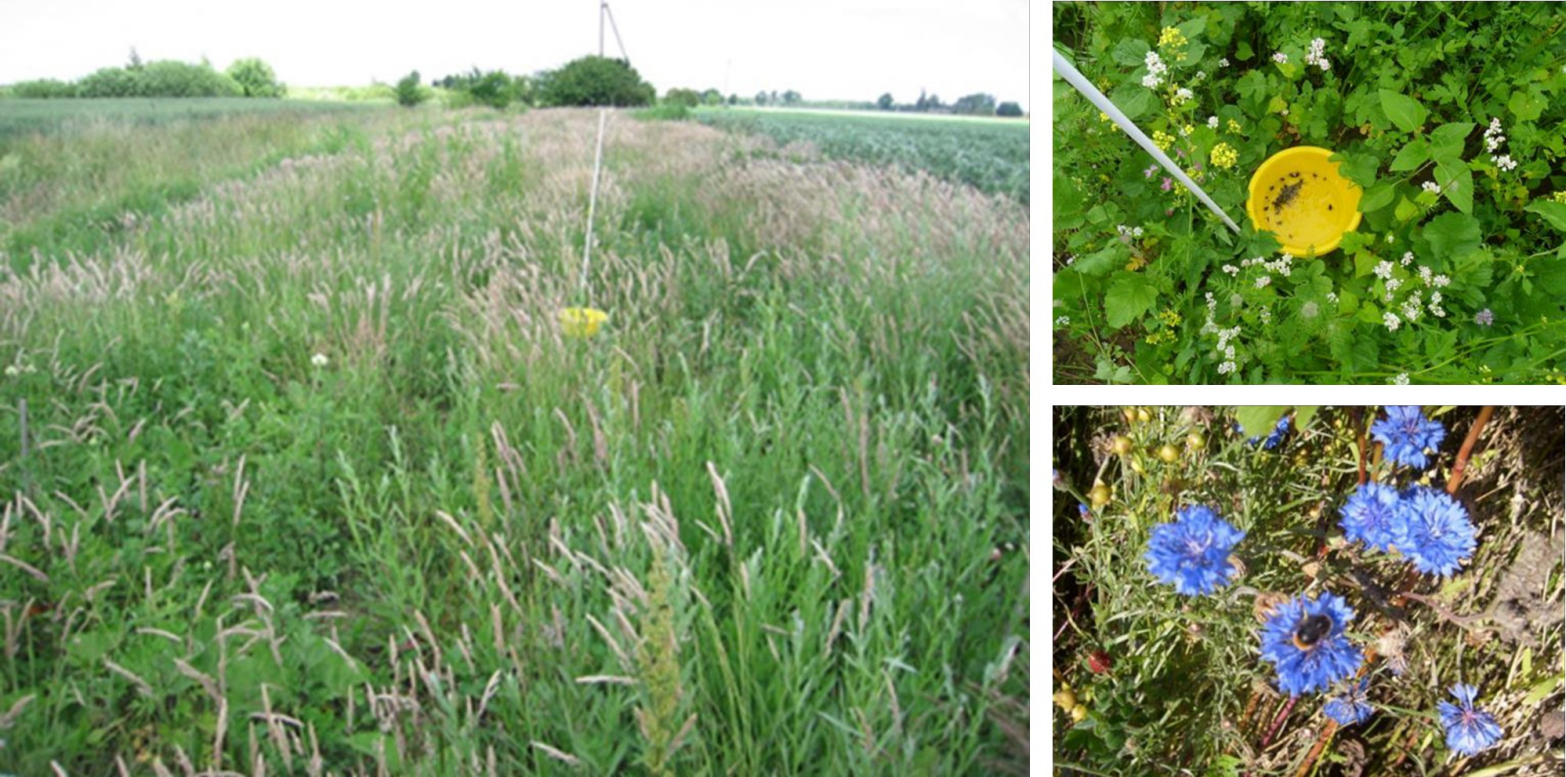
Plant edges strips on the in modern farming field.

*Experimental design and plant material*. In 2013 spring, the floral sward strips were established on the edges of large intensive farming land (field size > 5 ha). At the edge of the field, the measured strip is 360 m long and 6 m wide. The strip were spited into 4 parts (80 m x 6 m) and seeded with four plant combinations (mixtures): perennial grasses (PGS) as control treatment: *Festuca arundinacea* Schreb., *Festuca pratensis* Huds., *Festuca rubra* L., *Phleum pratense* L., *Dactylis glomerata* L., *Agrostis capillaris* L., (seed rate 20 kg ha^-1^); perennial legumes (PLS): *Trifolium pratense* L., *Onobrychis viciifolia* Scop., *Medicago sativa* L., *Trifolium repens* L., *Lotus pedunculatus* Cav., (seed rate 20 kg ha^-1^); annual dicotyledons (AFP): *Helianthus annuus* L., *Fagopyrum esculentum* Moench, *Borago officinalis* L., *Sinapis alba* L., *Phacelia tanacetifolia* L., *Linum usitatissimum* L., *Lupinus luteus* L., *Melilotus albus* L., *Trifolium resupinatum* L., (seed rate 33 kg ha^-1^); natural grassland (NGS): the base cover was formed from grasses: *Poa palustris* L., *Poa compressa* L., *Corynephorus canescens* L. *etc*.; perennial legumes: *Trifolium rubens* L., *Medicago lupulina* L., *Vicia cracca* L. *etc*.; flowering meadow plants: *Centaurea jacea* L., *Agrimonia eupatoria* L. *etc*., (seed rate 16 kg ha^-1^). The seeds of species of flowering plants from semi-natural swards (Fig 1) were collected during expeditions (in the first half of September 2013) in meadows of rivers’ valleys. Lithuanian varieties or their forms were used in other mixtures. The sward was cut once in sowing year. After the sward growth years (in 2014, 2015, 2016, 2017 and 2018) in September, the aboveground mass was cut and removed from the field, leaving tall stubble and plant fall. The annual plant mixture was seeded annually with being shallow tilled by 10–12 cm depth in late autumn for this treatment. In a modern field edge, where floral sward strips were established, winter wheats, winter wheats, rapeseed and winter wheat were grown during experimental period.

#### Plant analysis

At the end of the plant growing season (in September) the aboveground mass of perennial/annual plants were measured. Sampling of aboveground mass of four randomly chosen squares of 0.25 m^2^ in each strip were cut to ground level, weighed and dried. To determine the roots mass and residues of plants, monoliths were dugged out in the plots of ach treatment at a depth 0–25 and 0–50 cm (in the same place one after the other), using metal frame in size 25 x 25 x 24 cm. The large roots were separating from the soil of the monoliths, washed and dried. The fine roots and plant remain were separating by washing the soil through a sieve with a mesh size of 1 mm and dried. After plant mass was weighed, the dry matter was determined (dried to constant mass at 105°C) and the aboveground and belowground mass (roots and plant residues >1mm) of perennial/annual plants was calculated. The samples of soil and monoliths were taken from each strip with three replications.

#### Soil analysis

Soil samples for the determination of chemical characteristics (organic carbon, total nitrogen and mobile phosphorus and potassium) collected in strip and winter wheat field (50 distance from strips) soil at the 0–25 and 20–50 cm depth layers in the last experimental year (2018 end of August). Analysed soil samples were air-dried at room temperature, visible roots and plant residues were manually removed, crushed and sieved through 2 mm mesh and homogeneously mixed. After wet combustion organic carbon (SOC) was determined by a spectrophotometric measurement at 590 nm (UV/Vis Cary 50, Varian Inc., Palo Alto, CA, USA) with glucose as a standard [19]. The content of total nitrogen (N_total_) was determined after the wet digestion process with sulfuric acid (H_2_SO_4_) by the Kjeldahl method, using a spectrophotometric measuring procedure (UV/Vis Cary 50, Varian Inc., Palo Alto, CA, USA) at the 655 nm wavelength [20]. The samples were analysed for mobile phosphorus (P_2_O_5_) and potassium (K_2_O) by Egner-Rim-Doming (A-L) method. All soil concentrations of elements and compounds are expressed on DM basis after samples were dried to a constant weight at 105°C in a forced-air oven.

For the determination of soil physical properties samples were collected (at the depth 0–30 cm) in the year of field experiment upon completion. Soil moisture was measured in the end of plant vegetation at 0–30 cm depths by drying soil samples at 105°C for 48 h. Undisturbed core samples (5 cm length, 5 cm in diameter) were taken for soil bulk density and porosity was determined using stainless steel rings (100 cm^3^ volume) before crop harvesting at the 0–5, 5–10, 10–15, 15–20, 20−25, 25–30 cm depths. The averaged results of these indicators are presented in the article of 0–30 cm depth. The cores were oven-dried at 105°C for 48 h. The data were averaged for the 0−30 cm depths. Soil total porosity was calculated as follows: Ptv = (1–D / Ds) × 100, where Ptv is soil total porosity, % (v/v), D–soil dry bulk density, Mg m^-3^ and Ds–density of solid soil phase, Mg m^-3^. For distribution of the size of aggregates and water-stable aggregates (WSA), the soil was sampled at 0–15 and 15–30 cm depths. The soil was air-dried and then sieved into eight size fractions 8.0 mm diameter using a Retsch sieve shaker. The structure of the macroaggregates was determined and the structural factor was calculated as a ratio, the amounts of 0.25–5 mm soil structural aggregates size (%), and aggregate < 0.25 mm and > 5 mm (sums %). Formation of water-stable aggregates (WSA) was determined by using a wet sieving apparatus (Eijkelkamp Agrisearch Equipment, the Netherlands). Four replications of 4 g of airdried soil aggregates (1−2 mm size) were wet sieved (0.25 mm mesh size) in distilled water and then stable aggregates were destroyed by 0.2% NaOH solution, oven-dried at 105°C for 24 h and weighed [21].

**Fig 2:**
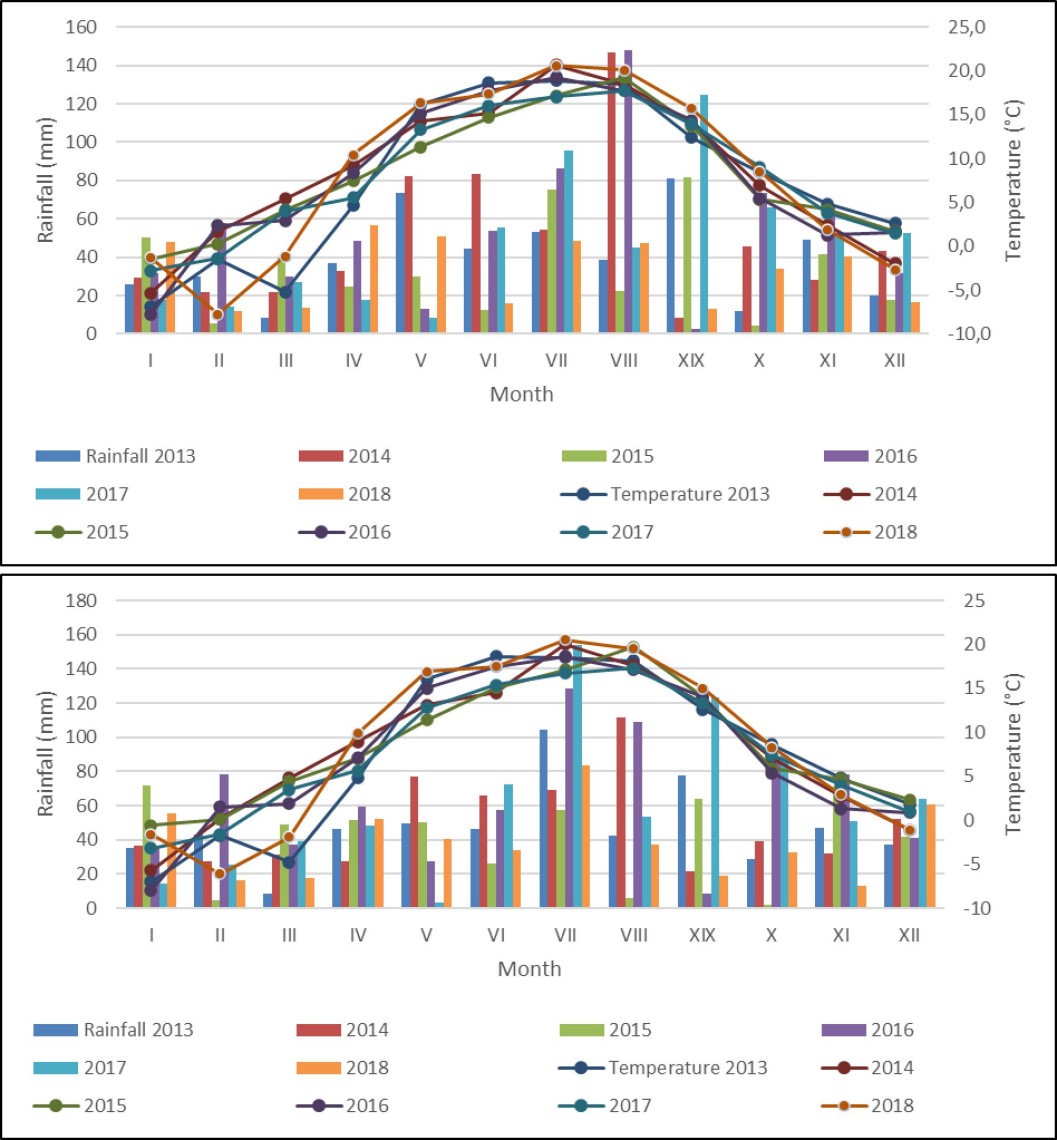
The mean annual air temperature and precipitation during the growing seasons in 2013–2018.

In the Central Lithuania’s territory, the precipitation falls enough (400 to 750 mm), but the low thermal conditions (active temperatures amount ∑t > 10° C = 2060–2200) is the most important limiting factor for the plant growth. The mean annual air temperature in the Joniškėlis is 8.1°C and in the Akademija– 7.9°C. In 2013 and 2018, the temperature of the growing season was higher than the long-term average of 1928–2018 (Fig 2).

### Statistical analysis

Plant roots and residues, soil agrochemical properties data were subjected to a three-way analysis of variance (location, floral sward strips and soil layer) (ANOVA), whereas plant aboveground biomass and soil physical properties data were subjected to a two-way analysis of variance (location, floral sward strips). Before analysis, the datasets were checked for normality (Shapiro–Wilk test) and homogeneity of variance (Levene test). Three-way ANOVA was performed considering the following factors: location (Joniškėlis and Akademija), sward strips (PGS, PLS, AFP, NGS) and winter wheat, and soil layer (0–25 and 25–50 cm). Significant differences between factors and interactions were determined using the F-test at p < 0.05 and p < 0.01 probability levels. Significantly differences in data were calculated using Tukey’s studentized range test at p < 0.05, where means with the same letter were not significantly different. Standard error (SE) of the mean was used to represent error values and error bars. Individual correlations between SOC and plant root and residues mass were analyzed using Pearson correlations at the p < 0.05 and p < 0.01 confidence levels. Statistical analyses were performed using Statistica software, version 7.1 (StatSoft Inc., Tulsa, OK, USA) and Addinsoft XLSTAT 2022 (Long Island, NY, USA**).**

## Results and discussion

**Fig 3:**
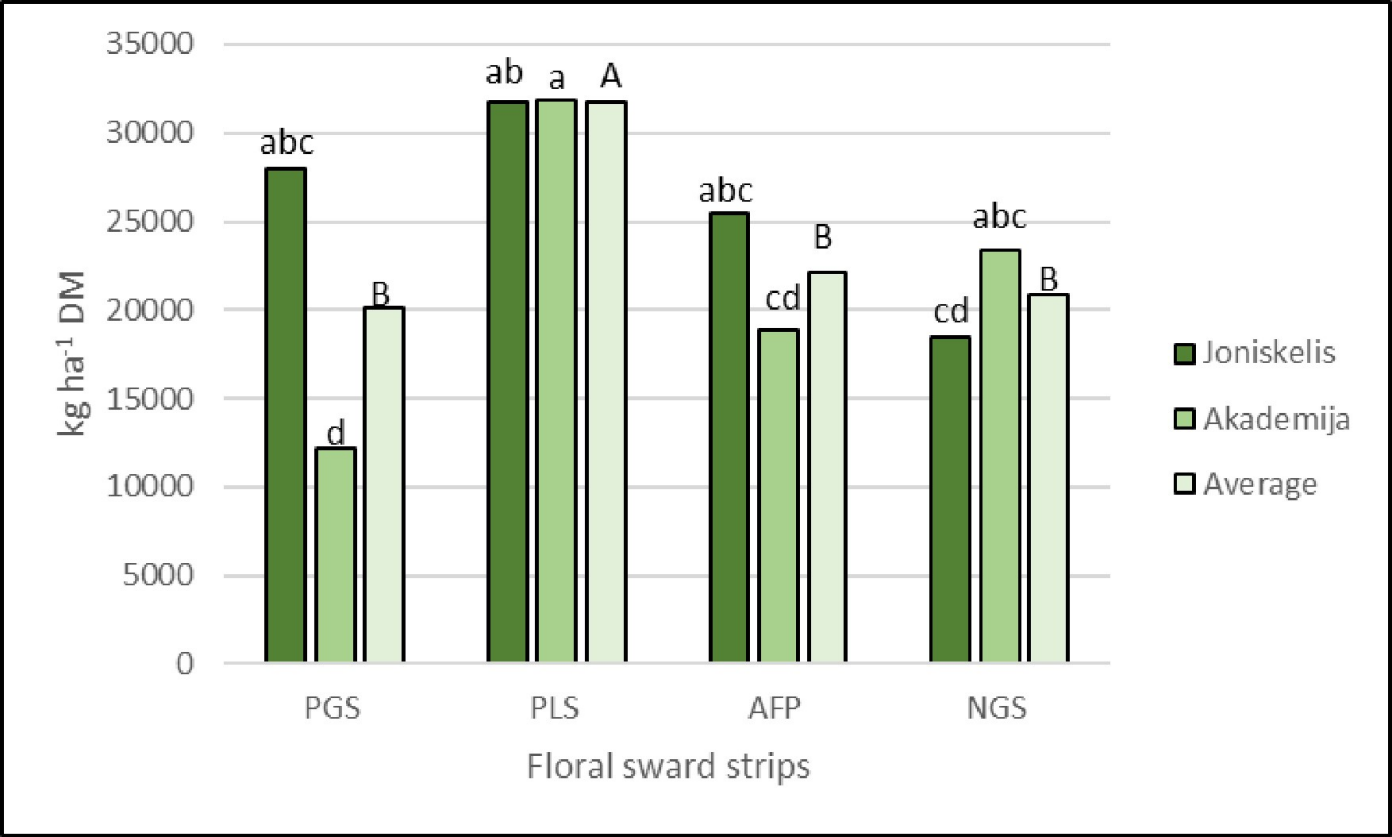
Total aboveground biomass of plants in a period of 5 years (2014–2018). Note. Perennial grass swards (PGS), perennial legume swards (PLS), annual floral plants (AFP) and natural grassland swards (NGS); means with different letters within individual interactions are significantly different at the *p* < 0.05 level.

Results of ANOVA showed that plant total aboveground biomass (sum of 5 year) was significantly influenced by interaction of location and sward strips (p < 0.05) (Table 1). In Joniškėlis (clay loam soil) site, the highest above-ground mass was found in PLS and PGS strips (31726.7 and 27944.2 kg DM ha^-1^ respectively), the lower–in NGS strip (18419.2 kg DM ha^-1^) (Fig 3). In this area, a significantly higher total aboveground mass yield (72.2%) was found for PLS compared to NGS. However, the data was not consistent across years. In Akademija (loam soil) site, the plant mixtures of strips developed slower compared to Joniškėlis due to different meteorological conditions. Significantly highest total aboveground mass was found at APL strip, compared to other strips. Here, the total five-year aboveground biomass was 31813.2 kg DM ha^-1^. During the research period, the lowest total aboveground mass was found in the perennial grass mixtures (PGS). Other researchers recorded the lowest production of aboveground mass at AFP (5810 kg ha^-1^), higher on PGS (6350 kg ha^-1^) [22].

**Table 1:** Probability (*p*) level of factors for plant aboveground and underground biomass (the table shows the sources of variation and probability for the *F*-test of each factor and its interactions).

Factor	Degree of freedom	Plant biomass, kg ha^-1^DM		
		Aboveground mass	Underground mass	
			Roots	Plant residues	Total
Location (L)	1/1	3,31	15.23	24.16	20.71
Floral sward strips (FS)	3/4	**5,30** *	12.12	7.43	11.00
Soil layer (SL)	1	-	**206.22** **	165.40	**233.50** **
L × FS	3/4	**3,60** *	1.18	1.71	0.45
L × SL	1	-	15.86	14.36	18.49
FS × SL	4	**-**	8.74	6.74	8.69
L × FS × SL	4	-	0.34	2.50	0.19

Note. 1/1– first number is aboverground mass, second number–underground mass

*—significant differences at 95% probability level

**—significant differences at 99% probability level.

Grasslands ensure along with the productive functions also significant non-production—ecological and environmental functions in agricultural landscape. The sod (root biomass and tillering zone), with a dominant share of 80% in primary grass swards production, ensures these functions. In all different plant mixes strips, the underground monoliths mass was accounted majority of the plant roots and plant residues. However, the amount of underground biomass accumulated by different flowering plant strips varied significantly. Joniškėlis the most soil enrichment with organic matter was found in the perennial grass swards (PGS) and natural grassland swards (NGS) strips, respectively 2,1702 kg ha^**-1**^ and 2,2074 kg ha^**-1**^, the plants roots and residues in these strips were significantly higher than the underground mass of other flower plant strips (Table 2). In the depth 0–25 cm of soil layer, the lowest amount of root and plant residues were found not only in the wheat field, but also in the cultivation of annual floral plants (AFP) and perennial legume swards (PLS) strips, respectively 12,142 kg ha^**-1**^, 11,131 kg ha^**-1**^ and 13,561 kg ha^**-1**^. The same tends emerged in the studies of other researchers, the lowest root biomass production was observed on AFP (7.30 t ha^**-1**^), the highest production on PGS (8.27 t ha^**-1**^) and amount of root biomass was significantly higher during the dry years than climatically normal and wet years [22]. In our results at Akademija site, the most soil residues with organic matter were found in the perennial grass swards (PGS) and natural grassland swards (NGS) strips. Plant roots and residues in the PGS strip were greater than the ground mass of the other plant strips. In the depth 0–25 cm of soil layer, the lowest amount of root and plant residues were found in the wheat field– 3420 kg ha^**-1**^. It was found that the highest amounts of roots and plant residues in the soil were left after cultivating grass strips of PGS and NGS compared to the field where cereals had been intensively grown. The highest amount of plant residues in the subsoil were found in the case of PLS and NGS. According to the average data the roots and plant residues were 11–12 times less in the soil depth 25–50 cm compared to the plough layer (0–25 cm).

**Table 2:** Dry mater of strip plant roots and residues mass (mean ± SE), kg ha^-1^.

Treat-ment	Soil layer, cm	Roots and plant residues					
		JON			AKD		
		root	plant residues >1mm	total	root	plant residues >1mm	total
Wheat	0–25	9461±1233.2a	2681±775.1a	12142±2008.6a	2497±971.2a	1402±495.1abc	3899±1458.9a
PGS		15147±1274.2bcd	6555±691.4b	21702±583.4c	10945±4338.8bcd	3281±1133c	14227±5420.2bcd
PLS		10189±584.3ab	3372±238.4a	13561±822.7a	5606±1263.7ab	2380±1243.9bc	7985±2460.2ab
AFP		7786±286.1a	3345±168a	11131±454.1a	5796±640.1ab	134±8.1a	5929±642.8a
NGS		19024±2928.3d	3050±213.6a	22074±3141.9c	12750±1237.9d	2699±460.1bc	15449±1013.5d
Wheat	25–50	461±31.8a	155±22.2a	616±54a	127±67.1a	43±4a	170±69.7a
PGS		874±140.6abc	275±14.1ab	1149±154.4a	342±223.7a	82±56.5a	424±280.2a
PLS		1667±197.5abc	374±13.9b	2041±211.3abc	317±85.4a	218±34.6b	534±561.4a
AFP		641±54a	150±0.3a	790±53.7a	2923±832.5c	55±15.2a	2978±819.2c
NGS		2109±867.2c	631±123.6c	2740±990.7c	2285±263.6c	10±3.1a	2295±262.4c
Soil layer (SL)							
*Average 0–25*					9920A	2889.9A	12810A
*Average 25–50*					1175B	199.2B	1374B

Note. Perennial grass swards (PGS), perennial legume swards (PLS), annual floral plants (AFP) and natural grassland swards (NGS); SE—standard error; means with different letters within individual interactions are significantly different at the *p* < 0.05 level.

## Soil physical properties

Soil analyses of flowering plant strips carried out in the last experimental year to evaluate differences of soil parameters in flower strips and their influence on edge of soil properties were intensively growth wheat. Various soil physical properties traits were analysed using statistical analysis of variance (ANOVA) in a two-way dataset, where the factors were location and swards strips. The data in Table 3 shows that the location was a primary source of variation for bulk density, porosity and structural coefficient. Stability of structural aggregates were influenced by the interaction of location and floral sward strips. Soil moisture did not depend on the studied factors.

**Table 3:** Probability (*p*) level of factors for soil physical properties in 2018 (the table shows the sources of variation and probability for the *F*-test of each factor and its interactions).

Factor	Degree of freedom	Moisture %	Bulk density, %	Total porosity, %	Structural coefficient	Stability of structural aggregates, %
Location (L)	1	0.08	61.84**	6.49*	29.80**	9.34*
Floral sward strips (FS)	4	1.05	1.10	0.43	1.93	13.82**
L × FS	9	0.16	1.18	1.93	3.56	17.46**

*Note*. *—significant differences at 95% probability level

**—significant differences at 99% probability level.

In both locations the highest moisture was performed in top-layer by growing strip of PGS, it has been affected by the resulting in turf. The moisture content was increased on average by 2.5 percent at the strip of grasses swards compared to wheat field soil (data not shown). In Akademija experiment site, the amount of soil moisture tends to increase by other swards strips as well. In clay loam site at Joniškėlis, the soil density was significantly (on average 6.9%) lower compared to the soil in Akademija (Table 4). The influence of the sward strips was ambiguous. In Joniškėlis site, margin sward strips tend to decrease soil bulk density. In loam site, the soil density was not significantly different from the control (winter wheat), it increased in the case where there was no grass in the mixture (PLS ir AFP). In clay loam soil the porosity was higher on average 5% compared to the loam soil. The growth of different plant species combination in the field strips had no significant effect for soil porosity.

**Table 4:** Influence of various plant strips on soil physical properties for the field edges (0–30 cm), (mean ± SE).

Treatment	Bulk density, %		Total porosity, %	
	JON	AKD	JON	AKD
Wheat	1.51±0.017bc	1.57±0.026ab	0.40±0.006ab	0.41±0.012ab
PGS	1.49±0.020c	1.57±0.007ab	0.42±0.006a	0.41±0.003ab
PLS	1.48±0.020c	1.63±0.032a	0.42±0.006a	0.39±0.012b
AFP	1.47±0.026c	1.62±0.023a	0.42±0.012a	0.39±0.009ab
NGS	1.47±0.003c	1.57±0.030ab	0.41±0.003ab	0.41±0.012ab
*Average*	1.48±0.008B	1.59±0.012A	0.42±0.003A	0.40±0.005B

Note. Perennial grass swards (PGS), perennial legume swards (PLS), annual floral plants (AFP) and natural grassland swards (NGS); SE—standard error; means with different letters within individual interactions are significantly different at the *p* < 0.05 level.

Soil structure formation and soil aggregate stability are affected by anthropogenic and natural environmental factors. Assessing the soil structure coefficient (the ratio of agronomically valuable and less valuable aggregates) it was found that the structure of loam soil was better and improved when growing perennial swards. The quality of the soil structure is defined by a coefficient whose value is close to >1. The structure of loam soil was better (structural coefficient was 29.5% higher) than that of clay loam soil and all swards had a positive effect on its structure (Table 5). The best soil structure was found when growing NGS and PGS (only in loam soil). The structural coefficient of clay loam soil was small (on average 0.74), it was mostly improved by NGS. Cultivation of various floral swards had an inconsistent effect on clay loam soil.

**Table 5:** Influence of various sward strips on soil structural indicators (0–30 cm), (mean ± SE).

Treatment	Structural coefficient		Stability of structural aggregates, %	
	JON	AKD	JON	AKD
Wheat	0.76±0.062cde	0.81±0.057cde	45.5±1.64f	69.8±1.18bcd
PGS	0.54±0.061e	1.26±0.034a	75.9±2.50bc	66.1±2.45de
PLS	0.78±0.069cde	1.06±0.207abc	73.0±1.06bcd	58.7±2.43e
AFP	0.69±0.013de	0.99±0.007abc	79.3±4.69ab	70.4±2.96bcd
NGS	0.91±0.095bcd	1.14±0.125ab	86.4±2.99a	67.0±4.69cde
*Average*	0.74±0.041B	1.05±0.058A	72.0±3.89A	66.4±1.59A

Note. Perennial grass swards (PGS), perennial legume swards (PLS), annual floral plants (AFP) and natural grassland swards (NGS); SE—standard error; means with different letters within individual interactions are significantly different at the *p* < 0.05 level.

It was determined that in clay loam soil all swards’ strips significantly increased the amount of stable structural aggregates (60.4–89.9%) compared to wheat soil. The highest amount of clay loam soil valuable structural aggregates was found in the soil of NGS strip. The loam soil (AKD) aggregate stability data showed an insignificant effect of grassland swards plants on the soil aggregates stability. Until now, we have little knowledge of the influence of nature grassland plants on soil properties.

The physical and mechanical properties of the soil are very different and largely depend on the amount of soil moisture in the field, the need for plants, and the meteorological conditions [23]. High soil aggregation is a significant soil quality parameter since it increases porosity, and therefore increases infiltration and water-holding capacity, and reduces erosion and runoff [24]. It is also known that soil aggregates stability increases with soil organic matter content and that soil management practices such as reduced tillage, crop residues and organic fertilizers can improve soil quality [25]. SOC, N_total_ and P_total_ had a positive effect on the formation of water-stable aggregates in different tillage [7]. SOM, clay minerals, bacteria, and fungi are important agents for the formation and stabilization of structural aggregates [26]. Silt and clay govern better binding of soil particles than sand. A high content of stable aggregates was indicated as an important factor in maintaining soil resistance to physical degradation [27].

## Soil chemical properties

The data in Table 6 shows that mostly soil agrochemical data were influenced by the interaction of factors. Soil layer and the interactions of location × margin sward strip had a significant effect on the variation of N_total_ content. The interactions of location × margin sward strip and the interactions of soil layer × location had a significant effect on SOC content. The interaction of all three factors had a significant effect on mobile phosphorus and potassium.

**Table 6:** Probability (*p*) level of factors for soil agrochemical properties in 2018 (the table shows the sources of variation and probability for the *F*-test of each factor and its interactions).

Factor	Degree of freedom	Agrochemical properties			
		N_total_ %	SOC, %	P_2_O_5_ mg kg^-1^	K_2_O mg kg^-1^
Location (L)	1	42.74**	0.06	7.96*	273.53**
Floral sward strips (FS)	4	2.56	8.94**	5.48**	3.59*
Soil layer (SL)	1	34.98**	105.12**	107.93**	253.77**
L × FS	4	4.07*	4.91**	5.51**	1.43
L × SL	1	0.02	4.88*	11.16**	32.36**
FS × SL	4	0.62	2.53	1.75	1.07
L × FS × SL	4	1.42	0.62	10.49**	4.40**

*Note*. *—significant differences at 95% probability level

**—significant differences at 99% probability level

The soil N_total_ depends on the enrichment of the soil with this element, the assimilation of N by growing plants and its migration through the soil profile. In addition, N interacts closely with SOC and soil biota. After comparing different soils in both experimental sites, it was found that the N_total_ concentration in clay loam soil (0–50 cm) was higher than loam soil (on average 43.3%) (Table 7). The studies have shown the N_total_ content increase in the clay loam soil by growing PGS, PLS, AFP and NGS strips compared with soil where winter wheat was grown.

**Table 7:** The effect of different herbaceous plant mixtures on the variation of soil total nitrogen and organic carbon (mean ± SE).

Treatment	N_total_ %		SOC, g kg^-1^	
	JON	AKD	JON	AKD
Interaction of location and floral sward strips (L x FS)				
Wheat	0.108±0.0112bcd	0.112±0.0135bcd	1.070±0.2043c	1.327±0.2205abc
PGS	0.162±0.0173a	0.110±0.0122bcd	1.672±0.1159ab	1.827±0.1945a
PLS	0.149±0.0143ab	0.076±0.0130d	1.643±0.1456ab	1.227±0.1790bc
AFP	0.141±0.0107abc	0.103±0.0124cd	1,453±0.1050abc	1.627±0.1801ab
NGS	0.135±0.0147abc	0.083±0.0098d	1.550±0.1589abc	1.308±0.1632abc
*Average*	0.139±0.0067A	0.097±0.0058B	1.478±0.0743A	1.463±0.0889A
Interaction of location and soil layer (L x SL)				
0–25 cm	0.158±0.0055a	0.115±0.0049b	1.718±0.0507a	1.835±0.0693a
25–50 cm	0.119±0.0100b	0.076±0.0081c	1.237±0.1097b	1.091±0.0901b
*Average 0–25*		0.137A		1.777A
*Average 25–50*		0.098B		1.164B

Note. Perennial grass swards (PGS), perennial legume swards (PLS), annual floral plants (AFP) and natural grassland swards (NGS); SE—standard error; means with different letters within individual interactions are significantly different at the *p* < 0.05 level.

In many cases N_total_ decreased in loam soil, however the differences were found not significant. Comparing the data between investigated margin strips, the highest amount of Ntotal was found in the PGS soil of both experimental sites. The value of this indicator declined in the deeper layer. In the deeper clay loam soil layer, the N_total_ concentration was 24,7% lower, o in loam soil– 33,9% lower compared to topsoil layer. There is difference were found significant. The increase of N_total_ in the soil can be linked to the immobilization of N into soil organic compounds [28].

Sward strips in field edges had the most positive influence on SOC (especially in clay loam soil). In clay loam soil (0–50 cm) the highest SOC was found in the PGS and PLS, the differences were essential (56.3% and 53.6% respectively) compared to the wheat soil. Floral strips did not differ significantly in terms of SOC. In loam soil experimental site, the accumulation of SOC was found highest amount in the PGS and AFP strips soil. Studies have shown that not only in top layer, but also in sub layer, there was a significant amount of SOC. The ratio of its quantities in top and sub layer was in clay loam– 1:0.72, in loam soil– 1:0.59 (in average). In loam soil, the highest difference of SOC was found between top and sublayer soil. The amount of SOC accumulated in the soil is determined not only by the amount of plant residues and roots, but also by their decomposition rate (C:N, chemical composition, biota activity). In clay loam soi (0–50 cm) the ratio of C and N (C:N) value was lower (C:N = 10.3–11.5) compare to the ratio in loam soil (C:N = 15.8–16.6). The C:N ratio slightly differed between the sward strips grown in field edges. However, the lowest value of the ratio C:N was found in the wheat soil.

**Fig 4:**
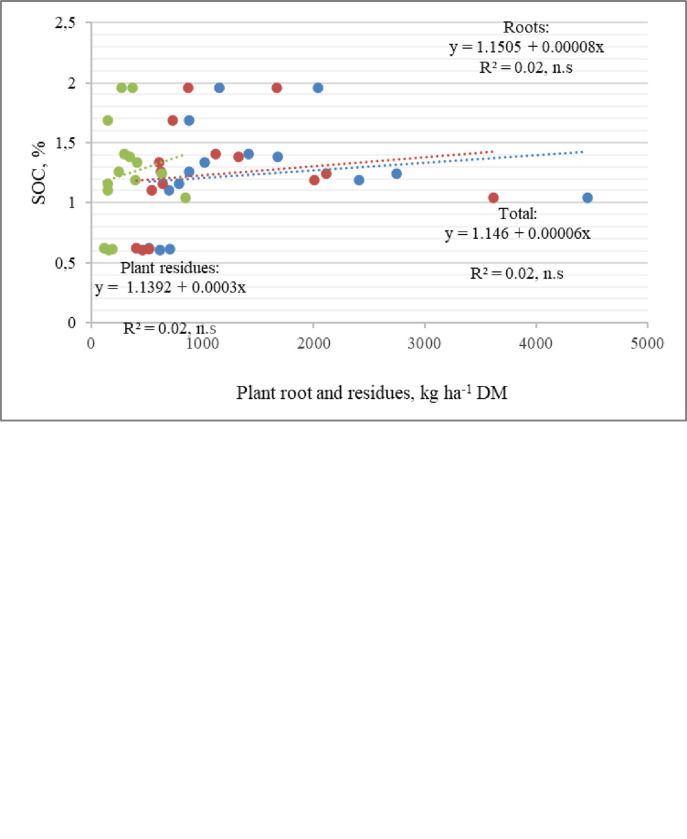
Effect of plant roots and plant residues on soil organic carbon content (Joniškėlis).

After performing a correlational regression analysis of the data, it was found that as the mass of plant roots and plant residues increased, the amount of SOC in the clay loam soil also increased. The correlation was not found in loam soil in Akademija site. In Joniškėlis site, the significant dependence was established when examining the roots and residues of plants at a depth of 0–25 cm (Fig 4A).

The content of SOC mostly depended on large roots, plant residues had no significant influence. In the deeper layer of clay loam soil, the content of SOC from plant roots and stems were found only increasing trends (Fig 4B). The data of Lithuanian colleagues showed that C transformation in the soil and chemical composition of the soil reflect this transformation, depending on soil management applied and the inherent properties of the soil itself. SOC has been increasingly considered as a key indicator of soil quality [25]. Literature sources claim that different types of clover–grass swards promoted SOC immobilization in microbial biomass and, at the same time, stabilized the sward ecosystem in the topsoil layer (soil respiration in 0–10 and 10–20 cm layers were lower) more effectively than the lucerne–grass sward [29]. Less intensive uses of lands, such as cover of grass [30,31] can be soil conservation techniques [23,32]. Over long periods, plant communities can impact through soil biota and abiotic properties [33–35]. Due to different soil characteristic, soils have different SOC storage potentials [36]. SOC is a significant binding agent that associates mineral particles together into aggregates [37]. Increasing the amount of organic carbon/humus in the topsoil and subsoil helps plants adapt to changing climate conditions (changes in temperature, moisture regime) or mitigate their negative effects [38].

In Joniškėlis location topsoil layer (0–25 cm), the concentration of P_2_O_5_ was significantly higher in PGS, PLS and AFP swards strips compared to wheat soil (Table 8). A significantly lower amount of mobile phosphorus was found in the NGS strip’s soil compared to the other investigated edge strips. In Akademija site, edge sward strips reduced amount of P_2_O_5_ in soil top layer compared to wheat. The highest concentration of P_2_O_5_ was found in the soil of PGS strips compare to others grown strips in field edges. The ratio of the amounts of mobile phosphorus in top and sub layer was 1:0.54 in clay loam and 1:0.70 loam soil (in average). In many cases, the edge sward strips reduce the amount of P_2_O_5_ in soil sublayer at 25–50 cm depth. In clay loam and loam subsoil the highest amount of mobile phosphorus was determined PLS strips. This resulted in the highest amount of roots and plant in the PLS soil sublayer. Phosphorus content in the wheat field could be increased by fertilization with P fertilizers.

**Table 8:** The effect of different herbaceous plant mixtures on the variation of soil mobile phosphorus and potassium (mean ± SE).

Treatment	P_2_O_5_ mg kg^-1^				K_2_O mg kg^-1^			
	JON		AKD		JON		AKD	
	0–25 cm	25–50 cm	0–25 cm	25–50 cm	0–25 cm	25–50 cm	0–25 cm	25–50 cm
Wheat	117±6.3bc	116±4.7bc	191±20.3a	97±12.4bcde	187±4.2d	138±4.0e	430±24.2a	207±30.4d
PGS	183±16.8a	60±5.6e	120±6.9bc	105±15.3bcd	204±11.0d	95±10.0e	309±14.2c	196±29.1d
PLS	176±5.5a	92±17.9bcde	99±1bcde	72±0.89de	201±7.9d	136±11.3e	389±35.4ab	192±10.3d
AFP	175±9.0a	72±6.5de	119±8bc	83±2.5cde	194±5.4d	118±6.6e	326±12.7c	217±10.8d
NGS	129±9.7b	77±27.5de	88±10.7cde	75±5.7de	193±8.1d	110±7.2e	352±25.5bc	189±8.4d
*Average SL*	156±8.2a	84±7.7c	123±10.5b	86±4.9c	196±3.3b	120±5.3c	361±14.8a	200±8.2b
*Average L*		120±8.7		105±6.6		158±7.7B		281±17.1A

Note. Perennial grass swards (PGS), perennial legume swards (PLS), annual floral plants (AFP) and natural grassland swards (NGS); SE—standard error; means with different letters within individual interactions are significantly different at the *p* < 0.05 level.

There was no correlation with the roots mass and plant residues. The data demonstrate that in less phosphorus-rich soil surface covered by swards–plants use phosphorus more efficiently and can prevent harmful phosphorus for migration to subsoil or groundwater. Land use is a main factor of nutrient loading to aquatic systems [39]. This is particularly the case in soils with severe granulometry, where high levels of clay particles are responsible for the negative properties of this soil.

The data showed that the migration of mobile P_2_O_5_ in loam soil was higher than in clay loam soil. The concentrations and movement of phosphorus in the subsurface soils influence different land uses and agriculture has high phosphorus leaching risk [40]. Also, plants can mobilize phosphorus from deeper soil layers and able store essential amounts until 70% of total phosphorus in the soil profile [41–43].

Soil available potassium is sensitive to environmental changes [44] and highly sensitive to the biochemical and biophysical processes of the plant [45]. The sites differed in the amount of mobile potassium in the soil–in loam soil (0–25 and 25–50 cm) it was more (77.8% in average) compared to clay loam soil. In Joniškėlis location K_2_O of all swards strips top layer increased, but not significantly compared to wheat soil. In the loam soil experiment, the value of mobile potassium (K_2_O) decreased compared to the wheat soil (in PGS and AFP and NGS significant). The most amount of K_2_O was accumulated in the topsoil of the PLS strip. The amounts of ratio mobile potassium in top and sub layer were 1:0.61 and 1:0.55 (in average) respectively in clay loam and loam soils. Subsoil chemical composition studies showed that a higher concentration of mobile potassium was in the PLS (Joniškėlis) and AEP (Akademija) strips compared to the other investigated field strips. Our studies show that the subsoil contains nutrients needed by plants and their reserves are not low.

According to other researchers, more than two-thirds of nutrients that can contribute to plant nutrition are found in the subsoil layer [46]. It is indicated that plants can absorb from the soil the nutrients they need from 10 to 70%. However, plant nutrition from the subsoil is not sufficiently studied and evaluated. While many studies have shown that soils low in phosphorus or potassium produce significant plant yields, other sources of nutrients (topsoil) have rarely been evaluated. The nutrient reserve in the subsoil is said to be particularly significant when the soil is dry or nutrient deficient (in low-input farming systems). In low-plant-diversity, intensive farming fields, the availability of nutrients from subsoil is limited due to higher soil density, lower oxygen content and microbiological activity, together with lower root length and density and the degree of mycorrhizal infection [12,47–49]. Studies clearly show that growth of perennial swards increases nutrient reserves in the subsoil layer. Nutrients amount depends on the types of cultivated plants (chemical composition of the residues), the activity of the soil biota, and the climate. The formation of biopores made by earthworms and the roots of deep-rooted plants, especially old ones–enriches the subsoil with nutrients from the topsoil layer and increases the penetration of the roots of plants grown after them into the subsoil and the availability of nutrients. Soil loosening with bio pores is said to be more sustainable than mechanical loosening [50].

## Conclusions

During the five-year period, the largest ground mass was grown by PLS. The highest amounts of roots and plant residues in the topsoil were left after cultivating plant strips of PGS and NGS compared to the field where cereals had been intensively grown. The highest amount of plant residues in the subsoil were found in the case of NGS. Such distribution of data was influenced by grasses swards. Bulk density, porosity and structural coefficient depended significantly on soil properties. Clay loam soil is more sensitive to mechanical compression than loam soil, so a more pronounced influence on the decrease in density and increase in porosity was observed here. The structure of loam soil was better than that of clay loam soil and all swards had a positive effect on its structure (especially NGS and PGS). However, in clay loam soil all swards’ strips significantly increased the amount of stable structural aggregates (60.4–89.9%) compared to wheat soil. Due to the large mass of plant residues and roots, the influence of swards on soil N_total_ and SOC accumulation is more pronounced in clay loam than in loam soil. Here, N_total_ significantly increased PGS, PLS. In clay loam soil, SOC significantly increased grown PGS, PLS in the field edge strips, and in loam soil–PGS (not significant) compared to the winter wheat field soil. The edge sward strips in clay loam soil toplayer increased the amount of mobile P, but at sublayer P_2_O_5_ decreased. In loam soil experimental site, the amount of P_2_O_5_ decreased in soil toplayer, and sublayer–little changed compared to the winter wheat soil. The amount of mobile potassium was significantly influenced by the soil and its layers. It can be stated that there are significant amounts of mobile P and K in sub layer (they accounted for 0.5–0.7 of their amounts in top layer).

## Supporting information

S1 File Fig 2 (a_b) Weather data. (Fig 2. The mean annual air temperature and precipitation during the growing seasons in 2013–2018) a) Location–Joniškėlis b) Location–Akademija.
(XLS)

S2 File Fig 3 data. Fig 3. Total aboveground biomass of plants in a period of 5 years (2014–2018).
(XLS)

S3 File Fig 4 primary data. Fig 4. Effect of plant roots and plant residues on soil organic carbon content (Joniškėlis) a) 0–25 cm depth b) 25–50 cm depth.
(XLS)

S1 Graphical abstract (PDF)
