# Supplementary material for: Effect of plant edges strips on the conservation soil properties in modern farming field
Source: PLoS One. 2024 Apr 16;19(4):e0299104. doi: 10.1371/journal.pone.0299104 (PMC11020931; doi:10.1371/journal.pone.0299104)
Supplement: S1 Graphical abstract — (PDF) [file pone.0299104.s004.pdf]

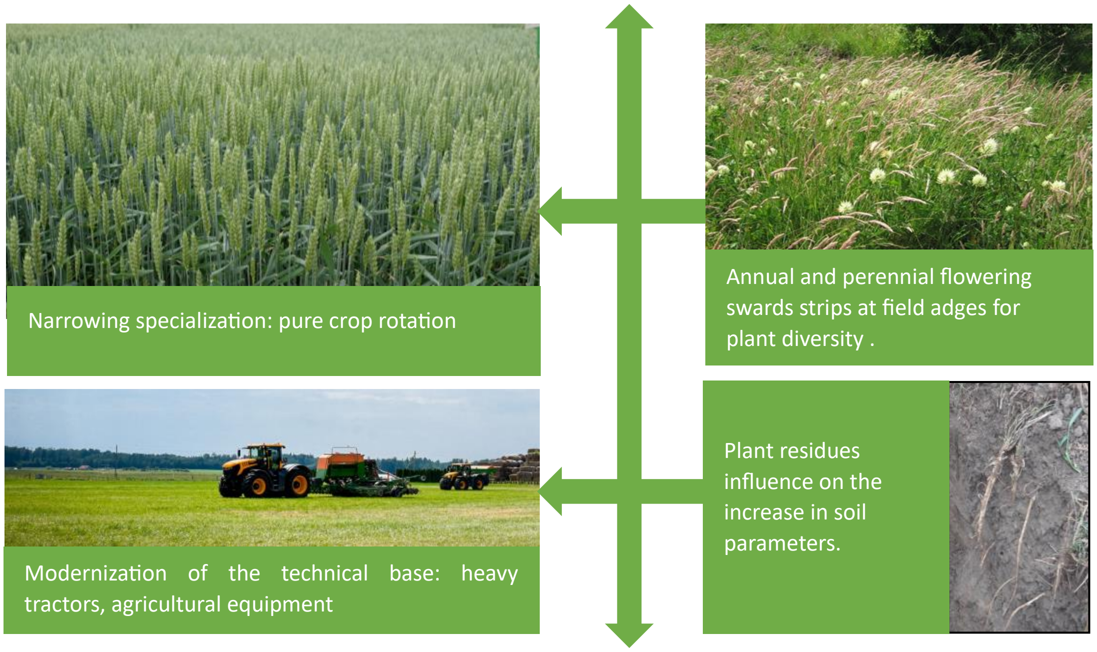
Narrowing specialization: pure crop rotation
Annual and perennial flowering swards strips at field adges for plant diversity .
Modernization of the technical base: heavy tractors, agricultural equipment
Plant residues influence on the increase in soil parameters.
